# Glenohumeral Joint Volume Measurement in Patients with Shoulder Instability: A 3D Volumetric Magnetic Resonance Arthrographic Study

**DOI:** 10.3390/medicina60091508

**Published:** 2024-09-16

**Authors:** Derya Güçlü, Elif Nisa Ünlü, Mehmet Arıcan, Oğuzhan Acar, Veysel Uludağ, Hayri Oğul

**Affiliations:** Faculty of Medicine, Duzce University, Düzce 81620, Turkey; nisaunlu@yahoo.com (E.N.Ü.); ari_can_mehmet@hotmail.com (M.A.); droguzhanacar@gmail.com (O.A.); vuludag1365@outlook.com (V.U.); drhogul@gmail.com (H.O.)

**Keywords:** atraumatic shoulder instability, shoulder capsule, joint volume, MR arthrography

## Abstract

*Background and Objectives:* This study aimed to compare capsular volume in patients with shoulder instability to that in control subjects without instability using magnetic resonance (MR) arthrography. The objective was to develop a reliable screening method with which to assess shoulder volume. *Materials and Methods:* In 21 patients with atraumatic shoulder instability and 21 controls, thin-slice 3D volumetric MR arthrography sequences were obtained. MR arthrography images were uploaded to 3D reconstruction, and 3D images were generated. From the 3D reconstructed images, volumetric measurements of rotator interval (RI), anterior and posterior capsular (AC, PC) recesses, biceps tendon sheath (BS), axillary recess (AR), and total glenohumeral joint (TGJ) were performed. Individuals with any extra-articular contrast leakage were also recorded. *Results:* A retrospective study analyzed a patient group of 21 individuals with shoulder instability (mean age 29.52 ± 12.83 years) and a control group of 21 individuals without instability (mean age 35.71 ± 12.77 years). No statistically significant differences were identified between the groups with regard to age, gender, or side distribution. The mean total joint volume was significantly higher in the instability group (29.85 ± 6.40 cm^3^) compared to the control group (23.15 ± 3.48 cm^3^, *p* = 0.0001). Additionally, the mean volumes of the RI, AC, PC, BS, and AR were all significantly greater in the patient group compared to the control group. *Conclusions:* 3D volumetric MR arthrographic measurements of the shoulder joint capacity can provide valuable insights for clinical follow-up and guide surgical treatment decisions in cases of atraumatic shoulder instability.

## 1. Introduction

The shoulder is the joint in the human body with the greatest range of motion [1]. Shoulder instability (SI) is usually caused by repetitive trauma, congenital structural abnormalities, or overuse and can lead to pain, loss of function, and limitation of joint movement in patients [2,3]. The management and diagnosis of SI is often difficult, especially in complex cases such as multidirectional instability [4,5].

MR arthrography is an effective imaging method used in the diagnosis of SI. This method provides detailed information about intra-articular structures, allowing for accurate assessment of the total joint capacity and joint recess volumes [6]. Volumetric measurements with MR arthrography play a critical role in determining changes, especially in areas such as the rotator interval (RI), biceps tendon sheath (BS), and axillary recess (AR) [7,8]. In this study, volumetric analyses will be performed via the segmentation of various recesses of the joint capsule using MR arthrography in patients with atraumatic SI. Our aim is to understand the pathophysiology of SI and to obtain clinically relevant findings by analyzing the volumetric changes in the shoulder joint in detail. It is thought that these findings will make important contributions to the diagnosis and treatment of SI.

## 2. Materials and Methods

### 2.1. Patients

This retrospective study was conducted by reviewing the archived records of patients referred to our Radiology department for MR arthrography between October 2021 and November 2024. We examined all the reports, and patients with SI were included as the patient group. A control group, matched for age and sex with the patient group, was selected from patients who underwent MR arthrography for reasons other than instability. As a result of the review, 42 shoulder MR arthrograms were included in the study, with 21 in the patient group and 21 in the control group. Exclusion criteria included previous shoulder surgery, acute shoulder trauma, or inadequate MR arthrography for proper measurement. In addition, patients with a full-thickness tear of the rotator cuff or contrast extravasation into the subcoracoid or subacromial–subdeltoid bursae were excluded, as these conditions could alter the injected intra-articular volume. Patients with evidence of adhesive capsulitis in their clinical information or MRI arthrography were excluded from this study as this condition would decrease joint capacity. Approval for this study was granted by the local ethics committee (decision number: 2024/151).

### 2.2. Injection Technique and MR Arthrograms

For glenohumeral joint arthrography, diluted paramagnetic contrast media were prepared at a concentration of 1:200 (gadobutrol: Gadovist, Bayer, Germany). The injection was performed using a posterior approach under ultrasound guidance. The diluted gadolinium was administered via injection with minimal pressure until either plunger rebound was felt or the patient experienced pain, preventing further injection. At 10–30 min after the injection, MR arthrograms were acquired using a 3T MR imaging system (Magnetom Skyra; Siemens Healthcare, Erlangen, Germany) with a dedicated shoulder coil. Routine shoulder MR arthrography included sagittal oblique TSE T1-weighted imaging (TR/TE: 725/10; 4-mm slice thickness) and axial and coronal oblique fat-saturated TSE T1-weighted (TR/TE: 562/10; 3 mm slice thickness) sequences. Additionally, a T1-weighted 3D volume-scanning MR arthrography sequence (volumetric interpolated breath-hold examination or VIBE) (TR/TE: 7.76/3.62; 11° flip angle; 0.4-mm slice thickness) was obtained alongside the conventional sequences.

### 2.3. Image Analysis

All shoulder MR arthrograms were retrospectively reviewed together by two radiologists with 1 and 7 years of experience in musculoskeletal radiology, respectively, who were blinded to the patients’ clinical information. Image interpretation was performed after loading the MR arthrographic images onto an image reconstruction program (3D Slicer 5.6.2 software) and reconstructing 3D images of the glenohumeral joint. First, raw Digital Imaging and Communications in Medicine (DICOM) data of the VIBE images from the MR arthrograms were imported into 3D Slicer. The program automatically delineated 3D images by coloring the high signal area corresponding to the contrast material in the joint capsule. Capsular 3D volumes were then automatically measured with the same software.

From the reconstructed 3D images, volumetric measurements of the RI, AR, BS, anterior and posterior capsule (AC, PC), and total glenohumeral joint (TGJ), as well as any extra-articular contrast material leakage, were formed and noted for the patient and control groups by segmentation as described below.

**Rotator interval volume** measurements were taken from the anterior border of the supraspinatus tendon to the superior aspect of the subscapularis tendon at the level of the coracoid process. The base of the RI was defined from the superior aspect of the subscapularis tendon medially, extending from the base of the coracoid process laterally to the biceps pulley, as described in the study by Petchprapa et al. and Ogul et al. [8,9]. Since the boundary between the subcoracoid recess and the RI could not be clearly defined—especially in patients with instability due to the possibility of a tear in the capsule of RI—the volume of the subcoracoid recess was included in the RI volume for patients with effusion in the subcoracoid recess.

**Anterior and posterior capsular** volumes, which comprised the remaining joint volume excluding the AR, RI, and BS, were divided into anterior and posterior halves. The center of the glenoid served as the common origin for the vertical plane used to divide these volumes.

**Biceps tendon sheath volume** was measured from the upper margin of the biceps pulley superiorly to the bottom of the biceps sheath inferiorly.

**Axillary recess volume** was measured inferiorly from the superior to the inferior glenoid margin down to the inferior aspect of the axillary recess.

**Figure 1:**
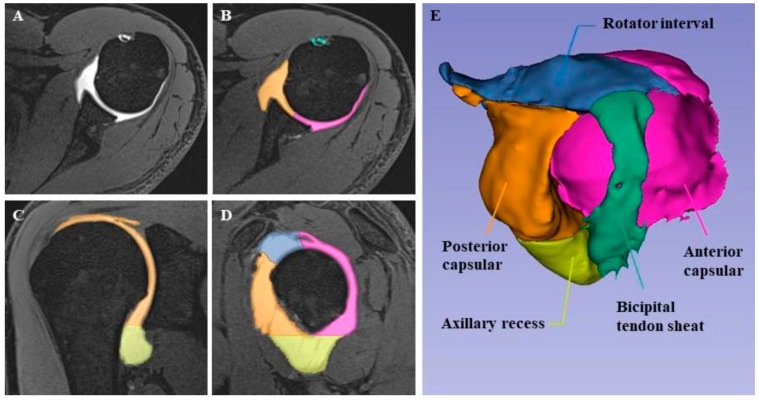
MR arthrography examination of a 35-year-old male patient with SI.

Additionally, the **total glenohumeral joint volume** was calculated and any **extra-articular contrast leakage** volume was recorded (Figure 1).

Above, axial (Figure 1A,B), oblique coronal (Figure 1C), and oblique sagittal (Figure 1D) fat-saturated 3D VIBE MR arthrography images show the magnetic resonance arthrogram with joint compartments in color. The 3D model (Figure 1E) was reconstructed using image processing software from MR arthrography images, showing the volumes of the glenohumeral joint and its components.

### 2.4. Statistical Analysis

In this study, statistical analyses were performed with the NCSS (Number Cruncher Statistical System) 2007 Statistical Software (Kaysville, Utah, USA) package program. In the evaluation of the data, in addition to descriptive statistical methods (mean, standard deviation), the distribution of variables was examined with the Shapiro–Wilk normality test; independent *t* test was used in the comparison of paired groups of normally distributed variables; chi-square test was used in the comparison of qualitative data; and Pearson correlation test was used to determine the relationship between variables. The results were evaluated at a significance level of *p* < 0.05.

## 3. Results

The retrospective study included a patient group of 21 individuals with atraumatic SI, 5 females (23.8%) and 16 males (76.2%), with a mean age of 29.52 ± 12.83 years. The control group consisted of 21 individuals without SI, including 7 females (33.3%) and 14 males (66.7%), with a mean age of 35.71 ± 12.77 years. There were no statistically significant differences in the mean age, gender, and side distribution between the patient and control groups (*p* = 0.125, *p* = 0.495, *p* = 0.537) (Table 1). Fifteen patients had anterior, five posterior, and two multidirectional instability.

The mean total joint volume was 29.85 ± 6.40 cm^3^ in the instability group and 23.15 ± 3.48 cm^3^ in the control group, with the patient group showing a significantly higher volume than the control group (*p* = 0.0001).

Additionally, the mean volumes of the RI, AC, PC, BS, and AR in the patient group were 6.00 ± 2.35 cm^3^, 8.59 ± 2.36 cm^3^, 9.25 ± 2.35 cm^3^, 1.29 ± 0.53 cm^3^, and 4.73 ± 2.90 cm^3^, respectively, while in the control group, they were 4.58 ± 1.53 cm^3^, 6.61 ± 2.06 cm^3^, 7.65 ± 1.46 cm^3^, 0.90 ± 0.38 cm^3^, and 3.41 ± 1.24 cm^3^. The volumes in the patient group were statistically higher than those in the control group (Table 2).

In patient and control groups, no statistically significant correlation was observed between age and volumetric RI, AC, PC, BS, AR, and TJV values (*p* > 0.05) (Table 3).

## 4. Discussion

In the present study, total glenohumeral joint volume and segmental glenohumeral volumes were all significantly increased in SI, which is in accordance with previous studies in the literature. An increase in joint capsular volume was demonstrated in MR arthrography in previous studies [3,10]. It is known that capsular laxity is the cause of recurrent shoulder dislocation, but no objective method has been established to diagnose SI either clinically or via imaging [11]. In the present study, we wanted to investigate the use of glenohumeral volume measurement via MR arthrography for the diagnosis of SI.

Several investigators have attempted to measure capsular laxity quantitatively in patients with SI using a variety of methods [11,12,13]. Some studies found that the capsular volume increased by determining the three-dimensional capsular volume with respect to the glenoid surface, and an increase in the sagittal cross-sectional capsular area was found to be related with SI [10,14]. On the other hand, a higher width and depth of the RI and a lengthening of the superior capsule were related with multidirectional atraumatic instability when compared with normal patients [14,15]. In Celantano’s and Provencher’s studies, RI width was found to be statistically similar between atraumatic SI and normal patients, where the RI dimension was defined as the shortest distance from the superior edge of the subscapularis tendon and the anterior edge of the supraspinatus tendon [16,17]. Also, some studies claimed that the main factors that lead to capsular laxity in SI are the lengthening of the inferior capsule and deficiency in the RI [15,18,19]. Lee et al. and Kim et al. also reported that inferior capsular dimensions and RI regions were significantly higher in SI patients compared with controls [19,20].

In previous studies, different measurement methods, like glenocapsular ratio or labrocapsular distance, were suggested for the detection of capsular laxity, and these studies are all in consensus about that the depth to which the axillary recess is associated with SI [10,21]. In accordance with previous studies, Celentano found that the width of the axillary recess at its largest point was significantly higher in patients with clinical SI compared the individuals without SI [16].

Dewing et al. studied the capsular area in SI and found that the posteroinferior capsular areas were larger in patients with posterior or multidirectional instability. In their study, anteroinferior and posteroinferior areas were determined on sagittal, oblique images where the capsular area is most distended by dividing the head of the humerus into two with a line that goes through the center of the humeral shaft [12].

In these studies, however, capsular laxity was assessed using two-dimensional (2D) images, which may not provide an accurate evaluation of the overall status of capsular laxity. Additionally, measurements were often taken from a single section, potentially leading to incomplete assessments. In contrast, we performed three-dimensional (3D) capsular volume measurements using a thin-section MR arthrography sequence (VIBE). This method appears to offer a more reliable and accurate estimation of the true glenocapsular volume.

Park JY et al. conducted anterior and posterior capsular volume measurements using computed tomography arthrography in their study. However, they did not provide detailed anatomical segmentation [22]. In the present study, we performed segmental volume measurements across five individual segments to identify differences in volume between segments. Our results showed an increase in volume in all segments among patients with SI compared to controls. This finding supports the notion that deformation occurs throughout all segments of the glenohumeral joint, rather than being confined to one or a few segments. We believe that this increase in volume reflects laxity and redundancy in the entire joint capsule, secondary to the viscoelastic properties of the capsuloligamentous structures.

Our study has some methodological limitations. First, the small number of patients included in the study limits the study’s generalizability. Second, it should be noted that the joint capsule volume varies between individuals. In this context, it is important to take into account differences in factors such as gender, height, weight, and body mass index, which vary according to the person concerned. Due to the retrospective nature of this study, patients’ height and weight were not assessed in this study. Another limitation is that the findings were not confirmed by an arthroscopic evaluation in the majority of the people in both groups. Finally, our controls were not completely healthy shoulders. However, in order to minimize this, we excluded shoulders with conditions in which the intra-articular volume may change as a result of shoulder pathology, such as a full-thickness tear in the rotator cuff.

Despite these limitations, to our knowledge, our study is the first to perform detailed segmentation and measure shoulder joint volume using MR arthrography in patients with instability, making it a significant contribution to the literature. Future studies with larger series and a prospective design including measurements such as patients’ height, weight, and body mass index will provide more comprehensive insights on this subject.

## 5. Conclusions

In conclusion, the results of this study suggest that identifying these findings on MR arthrography can support the diagnosis of multidirectional SI. Furthermore, this information can provide valuable insights for clinical follow-up and guide surgical treatment decisions.

## Figures and Tables

**Table 1:** Demographic data and distribution of shoulder side.

		Control Group		Patient Group		*p*
Age		35.71 ± 12.77		29.52 ± 12.83		0.125 *
Gender	Male	14	66.7%	16	76.2%	0.495 +
	Female	7	33.3%	5	23.8%	
Shoulder Side	Right	11	52.38%	9	42.86%	0.537 +
	Left	10	47.62%	12	57.14%	

* Independent *t* test; + chi square test.

**Table 2:** Comparison of the mean volumes between the patient and control groups.

	Control Group	Patient Group	*p*
Rotator interval (RI)	4.58 ± 1.53	6.00 ± 2.35	0.026 *
Anterior capsular (AC)	6.61 ± 2.06	8.59 ± 2.36	0.006 *
Posterior capsular (AC)	7.65 ± 1.46	9.25 ± 2.35	0.012 *
Biceps tendon sheath (BS)	0.90 ± 0.38	1.29 ± 0.53	0.009 *
Axillary recess (AR)	3.41 ± 1.24	4.73 ± 2.90	0.048 *
Total glenohumeral joint (TGJ)	23.15 ± 3.48	29.85 ± 6.40	0.0001 *

* Independent *t* test.

**Table 3:** Correlation between age and volumetric values.

		Age
Rotator interval (RI)	r	0.08
	*p*	0.613
Anterior capsular (AC)	r	−0.055
	*p*	0.732
Posterior capsular (PC)	r	0.201
	*p*	0.202
Biceps tendon sheath (BS)	r	−0.199
	*p*	0.206
Axillary recess (AR)	r	−0.353
	*p*	0.022
Total joint volume (TJV)	r	−0.074
	*p*	0.639

## Data Availability

The data is not publicly accessible due to privacy concerns but may be provided from the corresponding author upon request.
